# Bioproduction of Chitooligosaccharides: Present and Perspectives

**DOI:** 10.3390/md12115328

**Published:** 2014-10-28

**Authors:** Woo-Jin Jung, Ro-Dong Park

**Affiliations:** Division of Applied Bioscience & Biotechnology, Institute of Environment-Friendly Agriculture (IEFA), College of Agricultural and Life Sciences, Chonnam National University, Gwangju 500-757, Korea; E-Mail: woojung@chonnam.ac.kr

**Keywords:** bioproduction, chitooligosaccharides, chitin deacetylase, chitinase, chitosanase, enzymatic hydrolysis, transglycosylation, chemoenzymatic glycosylation

## Abstract

Chitin and chitosan oligosaccharides (COS) have been traditionally obtained by chemical digestion with strong acids. In light of the difficulties associated with these traditional production processes, environmentally compatible and reproducible production alternatives are desirable. Unlike chemical digestion, biodegradation of chitin and chitosan by enzymes or microorganisms does not require the use of toxic chemicals or excessive amounts of wastewater. Enzyme preparations with chitinase, chitosanase, and lysozymeare primarily used to hydrolyze chitin and chitosan. Commercial preparations of cellulase, protease, lipase, and pepsin provide another opportunity for oligosaccharide production. In addition to their hydrolytic activities, the transglycosylation activity of chitinolytic enzymes might be exploited for the synthesis of desired chitin oligomers and their derivatives. Chitin deacetylase is also potentially useful for the preparation of oligosaccharides. Recently, direct production of oligosaccharides from chitin and crab shells by a combination of mechanochemical grinding and enzymatic hydrolysis has been reported. Together with these, other emerging technologies such as direct degradation of chitin from crustacean shells and microbial cell walls, enzymatic synthesis of COS from small building blocks, and protein engineering technology for chitin-related enzymes have been discussed as the most significant challenge for industrial application.

## 1. Introduction

Chitin and chitosan have numerous applications as functional materials, as these natural polymers have excellent properties such as biocompatibility, biodegradability, non-toxicity, and adsorption. Chitin from crustacean shells is commonly obtained using inorganic acids for demineralization, with strong alkali for deproteinization [1,2]. These chemical processes have several drawbacks, including being a source of pollution [3] and reduction of depolymerization, and thus, chitin quality [4,5].

**Figure 1:**
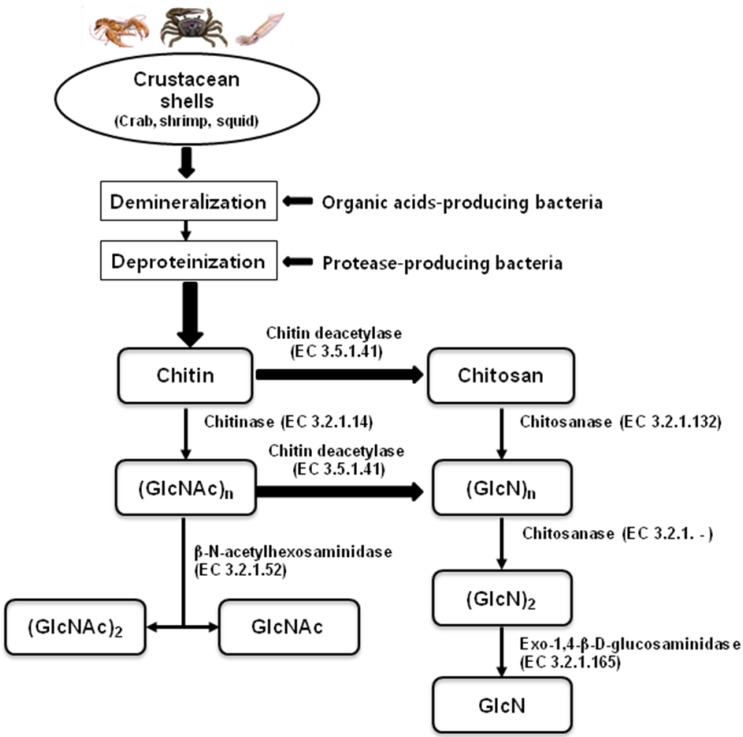
Flow chart for the bioproduction of chitin, chitosan, and their oligosaccharides from biological resources.

These chemical processes can be replaced by biotechnological processing with microbes and their metabolites, including organic acids and enzymes, as shown in Figure 1. The strains most frequently applied include *Lactobacillus* sp*.* and *Serratia marcescens* [6,7]. Biofermentation of crab shell wastes with 10% inoculums of *S. marcescens* FS-3 resulted in 84% deproteinization and 47% demineralization after 7 days of incubation [8]. Co-fermentation of the bacteria *Lactobacillus paracasei* KCTC-3074 and *S. marcescens* FS-3 [6] and the successive two-step fermentation with the two bacteria [7] provided more promising results for the production of chitin from crab shells.

Chitin and chitosan are high molecular-weight polymers with poor solubility at neutral pH values. This property limits their potential uses in the fields of food, health, and agriculture. However, these limitations may be overcome by the use of their oligomers or monomers. In humans, chitin monomers are precursors of the disaccharide units in glycosaminoglycans (such as hyaluronic acid, chondroitin sulfate, and keratin sulfate), which are necessary to repair and maintain healthy cartilage and joint function. Chitooligosaccharides (oligosaccharides derived mainly from chitin or chitosan, COS) have the potential ability to improve food quality [9,10,11] and human health [12,13].

COS mixtures can be prepared from chitosan by using different physical methods, like hydrothermal [14], microwave [15], ultra sonication [16] and gamma-rays [17]. Chemical methods using acid [18,19], H_2_O_2_ [20] or NaNO_2_ [21], can yield COS. Of chemical methods for hydrolysis of chitosan [18,19,20,21], acid hydrolysis is probably the best known. Early studies had shown that fully deacetylated chitosan is degradedto COS in concentrated hydrochloric acid [18]. In later studies [19], using a variety of chitosans, the acid-catalyzed degradation rates of chitosans were shown to depend on degree of deacetylation (DD). Acid hydrolysis was found to be highly specific to cleavage of GlcNAc-GlcNAc and GlcNAc-GlcN and A-D glycosidic linkages, with two to three orders of magnitude higher rates than the GlcN-GlcN and GlcN-GlcNAc linkages. In the same study, it was shown that the rate of deacetylation was less than one-tenth of the rate of depolymerization in concentrated acid, whereas the two rates were found to be equal in dilute acid [19]. Even though acid hydrolysis has been commonly used to prepare COS, these chemical and physical methods escape from the scope of this review and will not be dealt with more.

Enzymatic hydrolysis of chitin and chitosan has been proposed as an alternative method during the past few decades. Enzymes with hydrolytic activity on chitin and chitosan include chitinase, chitosanase, lysozyme, cellulase, pectinase, protease, lipase, and pepsin. These chitinolytic and chitosanolytic enzymes all have different modes of action and specificity for substrate size. A flow chart for the bioproduction of chitin, chitosan, and their oligosaccharides from natural resources by using enzymes and microorganisms is shown in Figure 1. Enzymatic hydrolysis seems to be generally preferable to chemical methods because the reaction is performed under more gentle conditions and the MW distribution of the product is more controllable [22,23]. However, production of well-defined COS in terms of length (degree of polymerization, DP), degree of de-*N*-acetylation (DD), and sequence (pattern of acetylation, PA) by enzymatic conversion processes is not straight forward [24]. The expensive cost of chitinases and chitosanases limits their wide application on an industrial scale, even using immobilized enzymes [22,25,26].

We are primarily concerned with the enzymatic hydrolysis of polysaccharides obtained from crustacean shells for the bioproduction of COS. In this review, we introduce the preparation and bioproduction of COS with enzymes from chitinase- and chitosanase-producing bacteria and fungi. Limitations and challenges in the bioproduction of COS are also discussed.

## 2. Bioproduction of Chitin Oligosaccharides and Its Monomer

Chitin oligosaccharides are produced from chitin by using chitinolytic enzymes. These enzymes hydrolyze the glycoside bonds between the sugars and are thus glycoside hydrolases (GH). GHs are classified in the Carbohydrate-Active enZYmes database (CAZy) [27,28,29,30]. The CAZy classification is purely based on the amino acid sequence similarity, which gives very useful information since sequence and structure, and hence mechanism, are related. In CAZy system, enzyme properties such as substrate and product activities, exo- *versus* endo-binding, processivity, and the presence of additional modules are not taken into account [27,28,29,30]. Chitinases occur in families GH18 and GH19 [31,32]. Chitinases have the unique ability to hydrolyze GlcNAc-GlcNAc bonds and this property discriminates these enzymes from chitosanases.

Chitinases can be classified into two major categories (endochitinases and exochitinases), according to their mode of action. First, endochitinases (EC 3.2.1.14; created 1961) cleave the linkage between GlcNAc-GlcNAc, GlcN-GlcNAc, and GlcNAc-GlcN in chitin chains to release smaller and more soluble chitin oligomers of variable size. Second, β-*N*-acetylhexosaminidase (EC 3.2.1.52; created 1972) includes subcategories of both chitobiase (EC 3.2.1.29; created 1961, deleted 1972) and β-d-acetylglucosaminidase (GlcNAcase, EC 3.2.1.30; created 1961, deleted 1992) [33]. Chitobiase catalyzes the progressive release of chitobiose starting at the nonreducing or reducing end of chitin. GlcNAcase progressively breaks down chitin polymer or chitin oligomers from the non-reducing or reducing end of the molecules, releasing β-d-GlcNAc or α-d-GlcNAc.These enzymes were prepared from bacteria and fungi, as shown in Table 1.

In COS production, it is practical to use crude enzyme preparations, such as microbial culture supernatants and their solid powders. Preparations of cellulase, protease, lipase, pepsin, chitinase, chitosanase, and lysozyme are mainly applied as commercial enzyme sources for the hydrolytic cleavage of chitin and chitosan (Table 1).

Pretreatment is necessary for enzymatic hydrolysis, because the strong crystallinity and insolubility of chitin in an aquatic environment. For example, chitin is normally treated with strong acids, such as hydrochloric acid (for colloidal chitin) [34] or phosphoric acid (for swollen chitin) [35] to break down crystal structure and increase the accessibility of the substrate to the enzyme. In contrast to chitin, chitosan is soluble in dilute mineral acids, thus forming salts with acids.

Enzymatic or chemical hydrolysis of chitin and chitosan results in a mixture of oligomers, not any specific oligomer, even with prolonged reaction time. Several techniques for separation and purification of COS have been reported, like gel filtration [36], ultrafiltration [37], and ion exchange [38] and metal affinity [39] chromatography. Preparative separation of COS is most commonly based on size, through size exclusion chromatography SEC). A SEC system using SuperdexTM 30 (GE Healthcare) columns, coupled in series, allowed separation of COS with similar DP values ranging from DP 2 to DP 20, independently of DD and PA [36]. Separation of COS can be achieved using cation-exchange chromatography, because protonated amino groups on the deacetylated sugars interact with the ion-exchange resin. With this method, COS of identical DP was separated based on the number of deacetylated units [38].

### 2.1. N-Acetyl-β-d-glucosamine

*N*-Acetyl***-***β**-**d-glucosamine (GlcNAc) was obtained with 85% yield from β- and α-chitin within 1 and 7 days, respectively, using chitinase from *Burkholderia cepacia* TU09 [40]. GlcNAc and *N,N′*-diacetylchitobiose [(GlcNAc)_2_] were obtained 75% and 20% yield from β-chitin within 6 days using chitinase from *Bacillus licheniformis* SK-1. In addition, chitinase from SK-1 produced GlcNAc with 41% yield from α-chitin. In most cases, β-chitin from squid pen is a better substrate than α-chitin from crab or shrimp shells.

GlcNAc and *N*-acetyl chitooligosaccharides were produced from colloidal α-chitin using a crude enzyme from *Paenibacillus illinoisensis* KJA-424 [7]. The production rate of monomer GlcNAc increased continuously during incubation, while that of GlcNAc oligomers declined. The maximum production of GlcNAc was 1.71 mg mL^−1^ (yield of 62.2%) after 24 h of incubation. At the same time (GlcNAc)_2_, tri-*N*-acetylchitotriose [(GlcNAc)_3_], hepta-*N*-acetylchitoheptaose [(GlcNAc)_7_] and octa-*N*-acetylchitooctaose [(GlcNAc)_8_] were 0.13 mg mL^−1^ (yield of 4.9%), 0.03 mg mL^−1^ (1.2%), 0.01 mg mL^−1^ (4.1%), and 0.24 mg mL^−1^ (9.6%), respectively.

Non-chitinase enzymes can be applied to the hydrolysis of chitin. GlcNAc was produced from α-chitin (from crab shell) and β-chitin (from squid pen) using crude cellulase preparations from *Trichoderma viride* (T) and *Acremonium cellulolyticus* (A) [41]. The yield of GlcNAc was enhanced by mixing cellulases T and A. Crystalline chitin (from bee) and α-chitin (from crab shell) were hydrolyzed with the enzymatic preparation Celloviridin G20x from strain *Trichoderma reesei*, which includes cellulases and β-glucanases [42]. After 10 days of incubation in these conditions, the yield of GlcNAc reached 86%.

The mixed enzymes (cellulase: lipase = 9:1) from *Aspergillus niger*, was used in various enzyme concentrations, to investigate the hydrolytic behavior on β-chitin [43]. Increasing substrate concentration while keeping the enzyme concentration constant resulted in higher GlcNAc yield. After 4 days of incubation, the yield of GlcNAc reached 61% from β-chitin (10 mg/mL).

Flake type of chitin together with swollen chitin, colloidal chitin, and powder chitin is also the substrate for the oligosaccharides production. GlcNAc was produced from crab shell α-chitin flake using crude enzyme extract from *Aeromonas hydrophila* H-2330, with 66%–77% yield after 10 days incubation at 17 °C [44].

Exo-type chitinases can use chitin oligosaccharides as a substrate. (GlcNAc)_2_ was gradually and completely degraded to GlcNAc by exo-type chitinase (*ChiA*71) from *Bacillus thuringiensis* subsp. *pakistani* with time [45] and by *N*-acetyl-β-hexosaminidase (*StmHex*) from *Stenotrophomonas maltophilia* [46]. GlcNAc was produced from (GlcNAc)_4_ by enzyme purified from *Aeromonas hydrophila* SUWA-9 [47].This kind of trial is not intended for the production of monomers, but for the elucidation of enzyme properties, as oligomers are much more expensive and valuable in application.

There are typical reports on the selective preparation of GlcNAc and (GlcNAc)_2_ [48]. It was found that α-chitin was effectively hydrolyzed to (GlcNAc) by *Aeromonas* sp. GJ-18 crude enzyme preparation below 45 °C. The enzyme preparation from *Aeromonas* sp. GJ-18 contained GlcNAcase and *N,N′*-diacetylchitobiohydrolase [49]. GlcNAcase was inactive above 50 °C, but *N,N′*-diacetylchitobiohydrolase was stable at this temperature. Therefore, GlcNAc and (GlcNAc)_2_ were selectively produced from α-chitin at two temperatures. At 45 °C, GlcNAc was the major hydrolytic product with a yield of 74% in 5 days’ incubation, while at 55 °C (GlcNAc)_2_ was the major product with a yield of 35% after 5 days’ incubation.

**Table 1:** Bioproduction of chitin oligosaccharides and its monomer by microbial enzymes.

Chitinsource	Enzyme Source	Enzyme	Mol. Wt.	Condition	Product & Yield	Analysis	Reference
Swollen chitin	*Aeromonas* sp. GJ-18	Crude enzyme	-	40 °C, 9 days	GlcNAc 94.9%	HPLC	[48]
						NH2P-50 4E	
Swollen chitin	*Aeromonas* sp. GJ-18	Crude enzyme	-	45 °C, 5 days	GlcNAc 74%	HPLC	[49]
					(GlcNAc)_2_ 4.8%		
				55 °C, 5 days	GlcNAc 3.9%	NH2P-50 4E	
					(GlcNAc)_2_ 34.7%		
α-Chitin	*Aeromonas* sp. GJ-18	Crude enzyme	-	Preincubation (50 °C, 60 min) 45 °C, 7 days	(GlcNAc)_2_ 78.9%	HPLC	[50]
β-Chitin					(GlcNAc)_2_ 56.6%	NH2P-50 4E	
α-Chitin	*A. hydrophila* H-2330	Crude enzyme	-	17 °C, 10 days	GlcNAc 64~77%	HPLC	[44]
(Flake & powder)						NH2P-50	
Chitin	*A. hydrophila* SUWA-9	Chitinase	-	37 °C, overnight	(GlcNAc)_2_~(GlcNAc)_5_	TLC	[47]
					GlcNAc		
Chitosan (60% DD)	*Bacillus cereus* TKU027	Chitinase	65/63 kDa	37 °C, 2 h 30 °C, 2 days	(GlcNAc/GlcN) DP 4~9	MALDI-TOF MS	[44]
		Culture supernatant			(GlcNAc/GlcN) DP 2~5		
β-Chitin	*Bacillus cereus* TKU022	Chitosanase	44 kDa	37 °C, 2 days	(GlcNAc)_2_, (GlcNAc)_4_	HPLC	[51]
					(GlcNAc)_5_, (GlcNAc)_6_		
Colloidal chitin	*Bacillus thuringiensis* subsp. *pakistani*	Exochitinase	66/60/47/32 kDa	37 °C, 24 h	GlcNAc	TLC	[45]
α-Chitin	*Burkholderia cepacia* TU09	Chitinase		37 °C, 7 days 37 °C, 1 days	GlcNAc 85%	HPLCNH2P-50	[40]
β-Chitin					GlcNAc 85%		
α-Chitin	*Bacillus licheniformis* SK-1	Chitinase		37 °C, 6 days	GlcNAc41%	HPLCNH2P-50	
β-Chitin					GlcNAc75%		
Swollen chitin	*Paenibacillus illinoisensis* KJA-424	Chitinase	38/54/63 kDa	37 °C, 24 h	GlcNAc 62.2%	HPLC	[7]
Chitin	*Trichoderma reesei*	Cellulases &β-glucanases	-	37 °C, 10 days	GlcNAc 86%	TLC/HPLC Separon SGX NH2	[42]
α-Chitin	*Trichoderma viride*	Cellulase	-	37 °C, 3 days	GlcNAc 16%	HPLC/NMR	[41]
	*Acremonium cellulolyticus*				GlcNAc 22%		
β-Chitin	Aspergillus niger	Cellulose & lipase	-	37 °C, 4 days	GlcNAc 61%	HPLC	[43]
						NH2P-50	
Chitin	*Thermococcus kodakaraensis*	Chitinase	90 kDa	70 °C, 3 h	(GlcNAc)_2_	TLC	[52]
	KOD1						
Chitin	*Vibrio anguillarum* E-383a	Exochitinase	-	-	(GlcNAc)_2_ 40.3%	HPLC	[47]
Chitin	*Enterobacter* sp. G1	Chitosanase	50 kDa	35 °C, 5 min	(GlcNAc)_2_	TLC/HPLC	[53]
Chitosan (80% DD)							
Chitin	*Corynebacterium* sp.	Chitobiase	-	40 °C, 24 h	GlcNAc	HPLC/NMR	[54]
Chitin, steam exploded	*Lecanicillium lecanii*	Chitinase		40 °C, 6 days	GlcNAcDP 1~9	HPLC/MALDI-TOF MS	[55]
(GlcNAc)_2,_	*Stenotrophomonas maltophilia*	*N*-acetyl-β-hexosaminidase & Chitin synthase		40 °C, 60 min	GlcNAc	HPLC	[46]
(GlcNAc)_6_							

### 2.2. N,N′-Diacetylchitobiose and Chitin Oligosaccharides

As mentioned above, (GlcNAc)_2_ can be obtained as a major hydrolytic product from enzymes by controlling the ratio of GlcNAcase to *N,N′*-diacetylchitobiohydrolase activities in the crude enzyme of *Aeromonas* sp. GJ-18 [50]. After 7 days of incubation, 78.9% and 56.6% of (GlcNAc)_2_ yields were obtained from swollen α-chitin and powdered β-chitin, respectively, using enzyme preparations that had been pretreated at 50 °C so as to inactivate GlcNAcase.

There are a few more reports on bioproduction of (GlcNAc)_2_. (GlcNAc)_2_ was produced from colloidal chitin by chitinase from *Vibrio anguillarum* E-383a, isolated from seawater [56]. The maximum yield of (GlcNAc)_2_ from chitin was 40.3%. (GlcNAc)_2_ was produced by shrimp α-chitin (100~200 mesh) from commercial bovine pepsin [57]. The yield of (GlcNAc)_2_ was 75%, while the yields of GlcNAc and (GlcNAc)_3_ were 19% and 9.5%, respectively. Tanaka *et al.* [52] also identified (GlcNAc)_2_ as an end product from colloidal chitin by using chitinase from the hyperthermophilic archaeon *Thermococcus kodakaraensis* KOD1.

The hydrolytic products of chitinase and GlcNAcase are mixtures of hetero-COS (DP 1–15), depending on reaction conditions. Hetero-oligosaccharides [(GlcNAc/GlcN) DP 2~5] were produced from chitosan (60% DD) by culture supernatant obtained from of *Bacillus cereus* TKU027. Chitin oligosaccharides [(GlcNAc/GlcN) DP 4~9] were produced by chitinase (65 and 63 kDa), and oligomers were identified using MALDI-TOF MS [58]. To analysis the distribution of chitin oligosaccharides, chitin oligosaccharides were derivatized with 9-aminopyrene-1,4,6-trisulfonate (APTS) and separated by capillary electrophoresis (CE) with laser-induced fluorescence (LIF) detection [59].

## 3. Bioproduction of Chitosan Oligosaccharides and Its Monomer

The most important tool in the biodegradation of chitosan to its oligosaccharides is chitosanolytic enzymes. The chitosanases have been prepared from various bacteria and fungi, as shown in Table 2. Chitosanases in glycoside hydrolase (GH) families 5, 7, 8, 46, 75, and 80 [27,28,29,30], have been classified into subclasses I, II, and III based on their substrate specificities toward chitosan [19,60,61,62,63]. Class I chitosanases can hydrolyze both GlcNAc–GlcN and GlcN–GlcN linkages, and class II chitosanases can split only GlcN–GlcN linkages, whereas class III chitosanases can degrade both GlcN–GlcNAc and GlcN–GlcN linkages. Chitosanases can also be classified into two major categories (endochitosanases and exochitosanases), according to their cleavage sites. Endochitosanases (EC 3.2.1.132; created 1990, modified 2004) cleave a partly acetylated chitosan at random and produces COS. Exochitosanases are usually called exo-1,4-β-d-glucosaminidase (GlcNase, EC 3.2.1.165; created 2008), which cleaves β-d-glucosamine (GlcN) residues continuously from the non-reducing end of the substrate ([33].

**Table 2:** Bioproduction of chitosan oligosaccharides and its monomer by microbial enzymes.

Chitosan Source	Enzyme Source	Enzyme	Mol. Wt.	Condition	Product & Yield	Analysis	Reference
Chitosan	*Bacillus* sp. KCTC 0377BP	Chitosanase	45 kDa	1700 (unit/mg)	(GlcN)_3_~(GlcN)_7_	TLC/HPLC	[64]
Chitosan	*Bacillus cereus* S1	Chitosanase	45 kDa	40 °C, 20 min	(GlcN)_2_ 27.2%	HPLC	[65]
					(GlcN)_3_ 40.6%		
					(GlcN)_4_ 32.2%		
Chitosan	*Bacillus* sp. 16	Chitosanase		37 °C, 30 min	DP 2~9 (DP 5~6)	TLC/HPLC	[66]
Chitosan	*Bacillus* sp. KFB-C108	Chitosanase	48 kDa	55 °C, 12 h	(GlcN)_3_~(GlcN)_5_	HPLC	[67]
Chitosan	*Bacillus* sp. HW-002	Chitosanase	46 kDa	40 °C, 5 h	(GlcN)_2_	HPLC	[68]
Chitosan	*Bacillus pumilus* BN-262	Chitosanase	-	45 °C, 1 h	(GlcN)_4_~(GlcN)_6_	HPLC (NH_2_-60) Bio-Gel P-4gel	[69]
					(GlcN)_5_~(GlcN)_7_		
Chitosan	*Bacillus megaterium* P1	Chitosanase (A/B/C)	43/39.5/22 kDa	28 °C, 12 h/90 h	(GlcN)_n_ oligomers	TLC	[70]
Chitosan	*Acinetobacter* sp. CHB101	ChitosanaseI (endo)	37 k Da	37 °C, overnight	>(GlcN)_5_	TLC	[71]
		Chitosanase II (endo)	30 kDa				
Chitosan (60% DD)	*Acinetobacter calcoaceticus* TKU024	Chitosanase (CHSA1)	27 kDa	37 °C, 30min	(GlcN)_n_ oligomers	-	[72]
		Chitosanase (CHSA2)	66 kDa				
Chitosan	*Nocardia orientalis* IFO 12806	Chitosanase (exo)	97 kDa (70 kDa)	40 °C, 24 h	GlcN	TLC/HPLC	[73]
Chitosan	*Matsuebacter chitosanotabidus* 3001	Chitosanase	34 kDa	30 °C, 10 min	(GlcN)_2_~(GlcN)_6_	TLC	[74]
Chitosan	*Aspergillus fumigatus* KH-94	Chitosanase I (endo)	22.5 kDa	50 °C, 30 min	DP 3~6 50% & >DP7 50%	TLC/HPLC	[75]
		Chitosanase II (exo)	108 kDa	50 °C, 5 min	GlcN		
Chitosan	*Aspergillus fumigates* S-26	Chitosanase	104 kDa	37 °C, 30 min	GlcN	TLC/HPLC	[76]
					(GlcN)_2_~(GlcN)_7_		
Chitosan	*Aspergillus oryzae* IAM2660	Chitosanase(endo) Chitosanase (exo)	40 kDa	37 °C, 20 min	>DP 5	TLC	[77]
			135 kDa	40 °C, overnight	GlcN		
Chitosan	*Trichoderma reesei* PC-3-7	Chitosanase	93 kDa	37 °C, 15 h	GlcN	TLC	[78]
Chitosan	-	Immobilized papain	-	-	MW < 10,000 49.5%	MALDI-TOF MS	[79]
					MW 600~2000 11.1%		
Chitosan(82.8% DD)		Novozyme lipase	-	37 °C, 24 h	DP 1–6	TLC	[80]
Chitosan (76% DD)	Commercial enzymes	Complex (cellulase, α-amylase, proteinase)	-	40 °C, 40 min	DP 5~17	MALDI-TOF-MS	[81]
Chitosan (95% DD)	*Bacillus cereus* TNU-FC-4	Chitosanase	46 kDa	45 °C, 33 min	>DP 7	HPLC	[25]
*Rhizopus oligosporus* cell wall	*Streptomyces* sp. N174	Chitosanase	-	40 °C, 24 h	(GlcN)_2_, GlcN-GlcNAc, (GlcN)_2_-GlcNAc	CP/MAS NMR/MALDI-TOF-MS	[ 82]
Chitosan	*Rhodothermus obamensis*	Branchzyme	256 kDa	50 °C, 24 h	DP 2–20	GC-FID/SEC-HPLC	[83]
Chitosan (60% DD)	*Penicillium janthinellum* D4	Chitosanase	49 kDa	50 °C, 60 h	DP 3–9	MALDI-TOF-MS	[84]

It is easier and more efficient to cleave the β-1,4-glycosidic linkages in chitosan than in chitin, because of its solubility in weakly acidic solutions. Chitosan, being a base, can be solubilized in both inorganic and organic acids, forming salts with acids. Acetic, lactic, and citric acids have all been used to facilitate enzyme hydrolysis. The solubility of chitosan depends on the molecular weight and degree of *N*-acetylation of the chitosan, concentration of the acid, and temperature. Homogeneous deacetylation of chitin to chitosan (approximately 50% DD) gives a water-soluble polymer.

The solubilized chitosan salt is a preferred substrate for chitosanases in homogeneous aqueous acids. Thus, chitosan is more rapidly hydrolyzed and gives a higher yield than chitin, which is not soluble and heterogeneous in aqueous environment. The products of the hydrolysis reaction of chitosan salt are accordingly the salt of its oligosaccharides. This means that a desalting process is necessary to obtain a pure oligosaccharide. Desalting is a rather complicated and costly process. In commercial products, the content has been expressed as being the salt of acetic, lactic, and citric acid of the oligosaccharides. Glucosamine is supplied in the marketplace as a dietary supplement in the form of a hydrochloric or sulfuric salt.

### 3.1. β-d-Glucosamine

The hydrolytic products of chitosan are mixtures of COS (DP 1–7), dependent on the reaction conditions, even though the monomer becomes a major end product after prolonged reaction time. Thus, chromatographic separation is necessary to purify each monomer, same as in chitin oligosaccharides. The enzymatic preparation of chitosan oligosaccharides is summarized in Table 2.

Koji mold *Aspergillus oryzae* is a strong producer of two different chitosanolytic enzymes, endochitosanase and exochitosanase (β-GlcNase). β-d-Glucosamine (GlcN) was produced as a final product from chitosan by a 135-kDa exochitosanase purified from *A. oryzae* IAM2660 [77]. In addition, chitosan oligosaccharides over DP 7 were produced from chitosan by a 40-kDa endochitosanase purified from *A. oryzae*. GlcN was produced from soluble chitosan by a 104-kDa exochitosanase purified from *A. fumigates* S-26 [76]. GlcN was produced from chitosan by a 108-kDa exochitosanase II purified from *A. fumigatus* KH-94 [75].

Chitosan oligosaccharides can be applied for chitosanase as a substrate, not for mass production of glucosamine but for elucidation of the reaction mechanism of chitosanase. GlcN resulted from the hydrolysis of chitobiose, chitopentose, and chitosan from the 79-kDa GlcNase of *Nocardia orientalis* IFO 12806 [73]. GlcN was also the final product from (GlcN)_6_ by the 93-kDa chitosanase purified from *Trichoderma reesei* PC-3-7 [78]. (GlcN)_6_ appeared to be hydrolyzed to GlcN_5_ and GlcN at the initial stage of the reaction.

### 3.2. Chitobiose and Chitosan Oligosaccharides

There are a few studies on the bioproduction of chitobiose (GlcN)_2_. Chitobiosewas produced from chitosan (75%~85% DD) in a mixture by chitosanase from *Bacillus cereus* S1 [65]. The composition of chitobiose, chitotriose, and chitotetraose was 27.2%, 40.6%, and 32.2%, respectively, after a 24-h reaction with a 45-kDa chitosanase. Chitobiose and chitotriose were produced from chitopentose and chitosan by crude proteins from *Acinetobacter* sp. CHB101 [71]. In the case of the 22.5-kDa endochitosanase purified from *Aspergillus fumigatus* KH-94, chitobiose, chitotriose, and chitotetraose were produced from chitohexaose [75]. In addition, chitosan oligomers of DP 3~6 (50% yield) and over DP 7 (50% yield) were produced from chitosan by the 22.5-kDa chitosanase.

These enzyme preparations contained endochitosanase activity. The endo-type activity is responsible for the production of chitosan oligosaccharides. Endo-type chitosanase is a major contributor to decreasing the viscosity of the reaction solution and fouling of the membrane reactor system [69]. The viscosity of high molecular weight material such as chitosan is involved in producing shearing forces in reactors, changes in enzyme-substrate affinity, and the fouling problem. When mixed with endochitosanase, the viscosity of chitosan decreased rapidly in accordance with production of the oligomers.

Thus, a great deal of interest has arisen regarding the reaction pattern of the endochitosanases. The chitosanase from *Bacillus* sp. KCTC 0377BP cleaved (GlcN)_6_ mainly into (GlcN)_3_ plus (GlcN)_3_ and to a lesser extent into (GlcN)_2_ plus (GlcN)_4_ [subsequently, (GlcN)_4_→(GlcN)_2_ + (GlcN)_2_] [64]. (GlcN)_7_ was cleaved into (GlcN)_3_ plus (GlcN)_4_ [subsequently, (GlcN)_4_→(GlcN)_2_ + (GlcN)_2_]. The purified endochitosanase (41-kDa) from *Bacillus cereus* D-11 hydrolyzed chito-oligomers (GlcN)_5–7_ into chitobiose, chitotriose and chitotetraose as the final products [85]. Minimal size of the oligosaccharides for enzymatic hydrolysis was pentamer. To further investigate the cleavage pattern of this enzyme, chitooligosaccharide alcohols (COS with the reducing unit converted into alditol) were prepared and used as substrates. The chitosanase split (GlcN)_4_ GlcNOH into (GlcN)_3_ + (GlcN)_1_GlcNOH, and (GlcN)_5_GlcNOH into (GlcN)_4_ + (GlcN)_1_GlcNOH and (GlcN)_3_ + (GlcN)_2_GlcNOH. The heptamer (GlcN)_6_GlcNOH was split into (GlcN)_5_ [subsequently,(GlcN)_5_→(GlcN)_3_ + (GlcN)_2_] + (GlcN)_1_GlcNOH, (GlcN)_4_ + (GlcN)_2_GlcNOH, and (GlcN)_3_ + (GlcN)_3_GlcNOH, whereas (GlcN)_1–3_GlcNOH were not hydrolyzed. The monomer GlcNor GlcNOH was never detected from the enzyme reaction. These results suggest that D-11 chitosanase recognizes three glucosamine residues in minus position and simultaneously two residues in plus position from the cleavage point [85].

Chitobiose as a main end product was produced from chitosan by a 46-kDa chitosanase purified from *Bacillus* sp. HW-002 [68]. A 34-kDa chitosanase purified from *Matsuebacter chitosanotabidus* 3001 was cleaved mainly (GlcN)_5_ and (GlcN)_6_ into (GlcN)_2_ plus (GlcN)_3_ [74]. Chitosan oligosaccharides [(GlcN)_2__–6_] were produced from chitosan by the purified chitosanase.

Pantaleone *et al.* [86] reported the hydrolytic susceptibility of chitosan to a wide range of enzymes, including glycanases, proteases, and lipases derived from bacterial, fungal, mammalian, and plant sources. These nonspecific enzymes have proven hydrolytic ability on chitosan to produce various chitosan oligomers in immobilized papain [79], lysozyme [87], and pronase [88]. Xie *et al*. [89] reported that chitosan (80% DD) was depolymerized by the cellulase of Aspergillus niger to give COS with DP 3–11. Mixture of cellulase, α-amylase and proteinasewas effective in the production of COS (DP 5~17) [81]. This finding presents another opportunity in the industrial scale production of COS, because these enzymes are active enough, commercially available, cheaper than chitosanase, and easy to handle. A commercial lipase was also applied for enzymatic preparation of COS, where COS with DP 1–6 was produced with 93.8% yield for 24 h hydrolysis at 37 °C [80]. Recently, the commercial α-amylase was used to hydrolyze chitosan from Clanis bilineata larvae skin under the optimal pH and temperature, thereby affording COS with a DP 2–8 [90].

Most chitosanase-producing bacteria require chitosan as a carbon/nitrogen source and chitosanase-inducer. Thus, one can get COS from the culture supernatant of the microorganism. Reducing COS sugars from shellfish waste were produced in the culture supernatant of *Acinetobacter calcoaceticus* TKU024, including chitosanases (CHSA1 and CHSA2) after 5 days of incubation [72]. Even though the yield of this method is very low, this kind of trial could be considered as a stepping stone to the direct extraction of COS from bio resources.

Intact cells of Rhizopus oligosporus NRRL2710were digested with a GH-46 chitosanase from Streptomyces sp. N174 [82]. Valuable hetero- and homo-oligosaccharides GlcN–GlcNAc, (GlcN)_2_–GlcNAc, and (GlcN)_2_ were produced, functionally, by the enzymatic digestion of the intact cells. The chitosanase digestion of intact fungal cells should be an excellent system for bioconversion of abundant microbial biomass without any environmental impact.

## 4. Chitin Deacetylase and Chitooligosaccharides

The chemical conversion from chitin to chitosan, the most difficult and cost-demanding step, has been done with 50% NaOH and high temperature in industrial applications. In nature, this step occurs at comparatively lower temperatures and neutral pH, by chitin deacetylase (CDA, EC 3.5.1.41; created 1976) [91]. The enzyme hydrolyzes the linkage between the acetyl group and the amine group in *N*-acetyl-d-glucosamine residues of chitin. Thus, bioconversion of chitin oligosaccharides to chitosan oligosaccharides can be achieved by CDA and *vice versa*.

Chitin deacetylase from *Absidia corymbifera* DY-9 was active towards water-soluble chitin (WSCT-50), glycol chitin, chitosan (DD 71%–88%), and chitin oligosaccharides with DP 2~7 [92,93]. CDA displayed little activity on chitin flakes, chitin powder, swollen chitin, or β-chitin powder. Chitin oligosaccharides were a comparatively good substrate because their solubility increased availability and accessibility for the CDA. Deacetylation rate on (GlcNAc)_2-6_ was size-dependent; greater lengths produced a higher rate of activity. These results suggest that solubilization of chitin is a limiting factor for enzymatic bioconversion of chitin to chitosan by CDA. Extracellular CDA from *Mortierella* sp. DY-52 was active on WSCT (DD 50%), glycol chitin and crab chitosan (DD 71%–88%) and also on *N*-acetylglucosamine oligomers (GlcNAc)_2–6_ [94].

Deacetylation by CDA is apparently substrate size-specific. GlcNAc was converted into GlcN by CDA from *Thermococcus kodakaraensis* KOD1, but (GlcNAc)_2_ was converted into GlcN-GlcNAc, neither GlcNAc-GlcN or GlcN-GlcN [95]. Only the non-reducing residue of (GlcNAc)_2_ has been deacetylated. CDA from *C. lindemuthianum* ATCC 56676 converted (GlcNAc)_2_ not into (GlcN)_2_ but into hetero-disaccharide GlcN-GlcNAc, and transformed (GlcNAc)_3_ and (GlcNAc)_4_ into the deacetylated products (GlcN)_3_ and (GlcN)_4_, respectively [96]. Alfonso *et al.* [97] found that chitosan oligosaccharides, (GlcN)_2-6_, were produced from chitin by the joint action of endochitinase and CDA from *Aspergillus nidulans*, suggesting that deacetylation mainly occurs after chitin oligosaccharide production by the endochitinase.

A solid natural substrate shrimp chitin could be deacetylated with an 11% deacetylation by CDA from *Saccharomyces cerevisia* [98]. Pre-hydrolysis of crystalline shrimp chitin by grape chitinases increased the deacetylation triggered by CDA and produced COS with a degree of deacetylation of 67% [98]. It is well known that the high crystallinity of chitin microfibril, by the hydrogen bond-stabilized packaging of chitin polymer, greatly impedes the access of the enzyme to the deacetylation reaction site in the chitin molecule. Enzymatic deacetylation is profoundly affected by the physical properties of the substrate, such as crystallinity, degree of deacetylation, particle size, and origin [99]. Pretreatment to destroy the crystalline structure prior to addition of the enzyme seems to be desirable, in order to improve the deacetylation rate and produce novel chitosan polymers and oligomers. The preparation of COS including *N*-deacetylation and transglycosylation are summarized in Table 3.

Martinou *et al.* [100] investigated the mode of action of CDA from *Mucor rouxii*on fully water-soluble partially *N*-acetylated chitosans (DP 30) and found that the CDA hydrolyzed acetyl groups according to a multiple attack mechanism, that is, CDA does not preferentially attack any sequences in the chitosan chains. Together with this, a multiple chain mechanism has been suggested in CDA originating from *Colletotrichum lindemuthianum* ATCC 56676 [99].

**Table 3:** Preparation of chitooligosaccharides by *N*-deacetylation and transglycosylation.

Product	Reaction Type	Reference
(GlcN)_2_~(GlcN)_6_	*N-*Deacetylation with endochitinase and CDA from *Aspergillus nidulans*; substrates: chitin	[97]
GlcN-GlcNAc	*N-*Deacetylation with CDA from *C. lindemuthianum* ATCC 56676; substrates: (GlcNAc)_2_	[96]
(GlcN)_3_ & (GlcN)_4_	(GlcNAc)_3_ and (GlcNAc)_4_	
Chitosan oligomers	*N-*Deacetylation with CDA from *Mucor rouxii*; substrates: partially *N*-acetylated chitosans (DP*_n_*, *n* = 30)	[100]
GlcN	*N-*Deacetylation with CDA from *Thermococcus kodakaraensis*KOD1; substrates: GlcNAc	[95]
(GlcN)_2_~(GlcN)_7_	*N-*Deacetylation with CDA from *Absidia corymbifera* DY-9; substrates: (GlcNAc)_2_~(GlcNAc)_7_ and WSCT-50	[94]
(GlcN)_2_~(GlcN)_7_	*N-*Deacetylation with CDA from *Mortierella* sp. DY-52; substrates: (GlcNAc)_2_~(GlcNAc)_7_	[91]
Chitosan (89% DD) & (GlcN)_2_~(GlcN)_4_	*N-*Deacetylation with CDA from *Saccharomyces cerevisiae*; substrates: Chitin and (GlcNAc)_2_~(GlcNAc)_4_	[98]
(GlcNAc)_6_~(GlcNAc)_15_	Transglycosylation reaction on β-1,4-(GlcNAc)_3_with lysozyme containing (NH_4_)_2_SO_4_ (30% w/v)	[101]
β-1,4-(GlcNAc)_2_ & β-1,6-(GlcNAc)_2_	Transglycosylation reaction on *N*-acetylchito-oligosaccharides [β-1,4-(GlcNAc)_2_ ~ β-1,4-(GlcNAc)_6_] with exo-β-d-GlcNase from *Alteromonas* sp. OK2607	[102]
*n*-Butyl β-d-glucosaminide (C4GlcN)	Transglycosylation reaction on chitosan oligosaccharides & *n*-butanol with exo-β-d-GlcNase from *Penicillium funiculosum* KY616	[103]

## 5. Transglycosylation and Chitooligosaccharides

In addition to hydrolytic activity, some chitinolytic enzymes possess certain level of transglycosylation ability, that is, the ability to transfer the released oligosaccharide moiety to a suitable acceptor to form a new glycosidic bond. The transglycosylation activity of these chitinolytic enzymes suggests great potential for the synthesis of size- and stereo-specific chitin/chitosan oligomers, or even polymers and their derivatives.

Preparation of higher DP chitin oligosaccharides was achieved by the transglycosylation reaction of glycolytic enzymes including chitinase, chitosanase, and other glycosidases. Chitinase purified from *Trichoderma reesei* KDR-11 was shown to convert (GlcNAc)_4_ into (GlcNAc)_2_(55.7%) and (GlcNAc)_6_(39.6%) by a transglycosylation reaction [104]. Akiyama *et al*. [105] reported that COS with DP 4–12 were successfully synthesized by a lysozyme-catalyzed transglycosylation reaction using *N*,*N′*,*N″*-tri(monochloro)acetylchitotriose and *N*,*N′*,*N″*-triacetylchitotriose as substrates followed by a base-catalyzed removal of the *N*-monochloroacetyl groups. Recently, Hattori *et al.* [101] made progress in biopreparation of COS. They tried lysozyme-mediated transglycosylation using β-1,4-(GlcNAc)_3_ as starting substance and successfully produced chitin oligomers of (GlcNAc)_6_ to (GlcNAc)_15_ in an aqueous system containing 30% (NH_4_)_2_SO_4_.

When GlcNase purified from *Penicillium funiculosum* KY616 was incubated with a mixture of chitosan oligomers and *n*-butanol, *n*-butyl β-d-glucosaminide (C4GlcN) was synthesized as a product by transglycosylation [103]. Yields of C4GlcN from chitobiose, chitotriose, and chitotetraose were found to be 14%, 23%, and 30%, respectively. The unusual β-1,6-(GlcNAc)_2_ were synthesized from β-1,4-(GlcNAc)_2–6_ by transglycosylation in chitinase preparation of marine bacterium *Alteromonas* sp. OK2607 [102].

Another example of enzymatic transglycosylation shows its diversity and potential in commercial applications. Fujimoto *et al.* [106] have reported the synthesis of gentiooligosaccharides (DP 3–9) from gentiobiose using a crude enzyme preparation from *P. multicolor.* The transglycosylation was shown to take place in two stages by a combination of β-glucosidase and β-(1-6)-glucanase. In the beginning, β-glucosidase produced gentiotriose from gentiobiose, and then β-(1-6)-glucanase acted on the resulting gentiotriose to produce a series of gentiooligosaccharides (DP 3–9) by transglycosylation. The transglycosylation reaction has high potential in the small-scale preparation of high value glycoside products applicable in medical and industrial fields.

## 6. Chemoenzymatic Glycosylation and Chitooligosaccharides

Chemoenzymatic glycosylation of chitooligosaccharides was intensively reviewed by the Kobayashi group [31,107,108,109]. *In vitro* synthesis of chitooligosaccharides was first reported by utilizingchitinase (EC 3.2.1.14) from *Bacillus* sp. (classified into glycoside hydrolase family 18; GH18) as catalyst [31,32]. On the basis of the transition state analogue substrate (TSAS) concept [31], the *N,N′*-diacetylchitobiose (GlcNAc-β-(1→4)-GlcNAc) oxazoline monomer was introduced. The enzymatic polymerization of this monomer proceeded via ringopening poly addition under weak alkaline conditions (pH 9.0~11.0), giving a synthetic COS with perfectly controlled stereo- and region selectivity. The DPs were evaluated as 10–20 depending on the reaction conditions [107].

The chitinase-catalyzed glycosylation using the sugar oxazoline substrate was applied to the stepwise elongation of GlcNAc unit by utilizing two enzymes, chitinase for ring-opening polyaddition of N-acetyllactosamine(Gal-β-(1→4)-GlcNAc) oxazoline (Gal = galactose) and β-galactosidase for the removal of the galactose unit from the produced oligosaccharide. [110,111,112,113,114]. The hydrolytic removal affords a new glycosyl acceptor with the GlcNAc unit at the non-reducing end. Repetition of these procedures using the two enzymes enabled the synthesis of chitooligosaccharides with desired chain lengths.

Interestingly, *N*-acetyllactosamine oxazoline was found to bepolymerized by chitinase A1 from *Bacillus circulans* WL-12 catalysis under basic conditions, giving rise to a novel oligosaccharide having the β-(1→4)-β-(1→6)-linked repeating unit in the main chain [115]. The DP of the resulting oligosaccharides was up to 5 based on the disaccharide. This was the first example of enzymatic glycosylation forming β-(1→6)-glycosidic linkage by chitinase catalysis.

The main disadvantage using the chemo-enzymatic approach is poor yield, because the product becomes necessarily a substrate for the enzyme (Table 4). Suppression of the chitinase-catalyzed hydrolysis of the product during the enzymatic polymerization is a challenge for chemoenzymatic glycosylation of COS. Wild-type chitinaseA1 from Bacillus circulans WL-12 has D202 and E204 residues as a DXE (D = Asp, X = any amino acid, E = Glu), a general sequence at the catalytic domain of chitinase [116,117]. A mutant chitinase E204Q (Q = Gln) exhibited less hydrolysis activity of the produced oligosaccharides by the enzymatic glycosylation, probably less protonation ability toward the oxygen of the glycosidic linkage by replacement of COOH in Glu with CONH_2_ in Gln [118].

**Table 4:** Chemoenzymatic preparation of chitin and chitosan oligosaccharides (COS) and its derivatives.

Product	Reaction Type	Reference
*N*-Acetylchitooligosaccharides	Chemoenzymatic elongation of *N*-GlcNAc unit by combined use of chitinase and β-galactosidase	[115]
Chitin derivatives with the deacetylated extents ranging from 0% to 50%	Chitinase-catalyzed copolymerization of *N-*acetylchitobiose oxazoline with the *N,N′*-diacetylchitobiose oxazoline	[54]
6- *O*-Carboxymethylated chitotetraose alternatingly	Chitinase-catalyzed chemoenzymatic glycosylation of 6- *O*- and 6′-*O*-carboxymethyled chitobioseoxazolines	[119]
3- *O*-Methylated chitotetraose	Chitinase-catalyzed chemoenzymatic glycosylation with 3- *O*-methyl- and 3′-*O*-methylchitobiose oxazolines	[118]
Oligo- *N*-acetyllactosamine derivatives with β-(1→4)-β-(1→6)-linked repeating unit	Chemoenzymatic polymerization by using transition state analogue substrate with chitinase A1	[116]
Alternatingly d-Glcβ-(1→4) *N*-GlcNAc repeating units, a cellulose-chitin hybrid polysaccharide	Chemoenzymatic glycosylation of Glcβ (1→4) GlcN Acoxazoline and GlcNAcβ (1→4) Glc fluoride by chitinase and cellulose, respectively	[103]
Alternatingly d-GlcNβ-(1→4) *N*-GlcNAc repeating units, a chitin-chitosan hybrid polysaccharide	Chitinase-catalyzed chemoenzymatic glycosylation of C-2′ position modified *N*-acetylchitobiose oxazolines	[55]
Fluorinated chitins	Chitinase-catalyzed polymerization C-6, C-6′, or both modified *N*-acetylchitobiose oxazolines	[120,121]

## 7. Perspectives

### 7.1. Direct Degradation and Separation of Chitin from Crustacean Shells or Squid Pens

The direct degradation and separation of α-chitin from crab and shrimp shells, and microbial cell walls poses a significant challenge. In light of the difficulties associated with traditional chitin oligosaccharides’ production processes, environmentally compatible and reproducible degradation alternatives are desirable. However, crustacean shells are not soluble in standard aqueous media and their crystallinity is potentially too high to be degraded enzymatically. To solve these problems, pretreatment to break the chitin crystal structure is widely considered.

In this regard, chitin substrates were pretreated with steam explosion prior to enzymatic reaction [122]. A 11.28% reduction of the crystallinity index was observed with steam explosion and a 23.6% yield of chitin oligosaccharides with DP up to 5 was achieved. Interestingly, Nakagawa *et al.* [123] reported a breakthrough in the direct production of oligosaccharides from chitin and crab shells. They introduced a combination protocol of mechanochemical grinding and enzymatic hydrolysis, and produced GlcNAc and (GlcNAc)_2_ directly from crab shells and chitin. The direct degradation ratio of the chitin in crab shell was close to 100%. For this purpose, they developed a novel “converge” mill [124], a derivative of the medium ball mill. Mechanochemical grinding, with the converge mill, was found to be extremely effective for pretreating chitin and crab shell before enzymatic digestion [125].

Breaking down the chitin crystal structure of biomaterials improves enzymatic degradation, allowing the enzymes to easily access and exert catalytic action. Wu and Miao [126] showed that mechanochemical treatment markedly increases the glucose yield from enzymatic corn flour hydrolysis. Similar results were observed by Fujimoto *et al*. [127] for lignocellulosic biomass. Van Craeyveld *et al*. [128] also showed improvement of extractability and molecular properties of *Psyllium* seed husk arabinoxylan by ball milling.

Mechanochemical grinding also provides an additional advantage. In the case of chitosan oligosaccharide production, salts are inevitably formed from aquatic reaction mixtures. Sometimes the bound salts limit application of COS. Salt production can be circumvented by mechanochemical grinding of the substrate, followed by enzymatic hydrolysis. 

### 7.2. New Enzymes with Better Properties

Solubilization has become the first choice for breaking down the chitin crystal structure in chitin and crab shells to improve enzymatic degradation. Solvents are necessary to solvate the substrate, and these are still being studied along with continuing research seeking enzymes that are stable in organic solvents or heterogeneous solvent systems. Enzymes that function in an extreme environment are important for this purpose and their mode of action differ fundamentally from of glycoside hydrolases.

During researches on enzymes capable of efficiently degrading recalcitrant polysaccharides such as cellulose and chitin for biofuels, it has been speculated about the existence of a substrate-disrupting factor that could make the crystalline substrate more accessible to hydrolytic enzymes [129]. Vaaje-Kolstad *et al*. [130] showed that CBP21 (CBP for chitin-binding protein), produced by the chitinolytic bacterium *Serratia marcescens*, is an enzyme that catalyzes cleavage of glycosidic bonds in crystalline chitin, thus opening up the in accessible polysaccharide material for hydrolysis by normal glycoside hydrolases. The products were identified as chitin oligosaccharides with a normal sugar at the non-reducing end and an oxidized sugar, 2-(acetylamino)-2-deoxy-d-gluconic acid (GlcNAcA), at the other end. This is an oxidative enzyme boosting the enzymatic conversion of recalcitrant polysaccharides. This finding helps understand the factors involved in degradation of crystallinechitin and the chitin cycle in nature and provides a stake to more precisely handle the process for COS production from crystalline chitin and even raw materials such as crab and shrimp shells.

Recently, several commercially available hydrolytic enzymes including lysozyme, cellulase, papain, pectinases, and hemicellulase were found to catalyze the cleavage of the glycoside bond in chitin and chitosan [83]. Use of these nonspecific enzymes together with specific chitinase, chotosanase, glycosyltransferase, and CDA certainly opens a possible route to optimize the hydrolysis reactions controlling the production of chitooligosaccharides. Although different nonspecific enzymes have been used to obtain COS from chitin and chitosan, due to the limited capacity of most hydrolytic enzymes there is still interest in finding new enzymes with better properties.

Branchzyme is a relatively inexpensive commercial preparation that contains a branching glycosyltransferase from *Rhodothermus obamensis* expressed in *Bacillus subtilis*. This enzyme catalyzes the transfer of a segment of a 1,4-α-d-glucan chain to a primary hydroxyl group in a similar glucan chain to create 1,6-α-linkages, thereby increasing the number of branch points [131]. Interestingly, Branchzyme was active on chitosan and produced COS with DP 2-20, with a higher concentration having COS with DP 3–8 [132]. Recently, a family 46 chitosanase from *S. coelicolor* A3(2) was employed to degrade both a fully deacetylated chitosan and a 68% deacetylated chitosan for the production of a series of COS and to study in-depth the enzyme's mode of action [133].

### 7.3. Manipulation of the Size-Distribution of Oligomers Produced by Enzymatic Bioconversion

The DD, DP, the molecular weight distribution, and *N-*acetylation pattern (PA) of the resulting COS mixture depend on the starting material (mostly DD of chitin and chitosan) and the specificity of the enzyme used. Product mixtures can be enriched for certain compounds by optimizing the substrate-enzyme combination. This is illustrated by several studies on enzymatic degradation of chitosans [134,135,136,137,138,139,140]. The degradation of chitosan by the family 18 chitinases, ChiA, ChiB and ChiC, from *Serratia marcescens* has been studied in detail [36,134,141,142]. The biphasic kinetics of the reaction with ChiB was illustrated, as in the initial rapid phase and the second much slower phase of the reaction [36]. Sorbotten *et al*. [36] showed that the size-distribution of oligomers could be manipulated by varying the DD of the chitosans (DD 87%, 68%, 50%, and 35%) with ChiB; the products get longer as the DD goes up. Very interestingly, Sikorski *et al*. [137,142] have produced a model for the degradation of different chitosans with ChiB, which is capable of predicting the length distributions and yield of the products after extended hydrolysis. These profiles allow for selection of optimal reaction and substrate parameters for efficient production of oligomers with desired lengths. Altogether, this suggests that production of oligomers with desired lengths can be achieved by manipulating the choice of the starting materials with specific DD, and PA, the choice of the enzyme, and the choice of the processing time [130].

### 7.4. Genetic Engineering Technology

Genetic engineering technology is applicable and promising for mass production of recombinant enzymes and understanding mode of action, which is useful for bioproduction of COS and synthesis of structure-specific oligosaccharides. Martinez *et al*. [51] declared that enzymatic synthesis of COS has been a matter of research by exploiting the transglycosylation activity of retaining family 18 chitinases. The mutagenesis of two GH-18 glycoside hydrolase, B. circulans WL-12 chitinase A1 (Bc ChiA1) and Trichoderma harzanium chitinase 42 (Th Chit42) abolished the hydrolytic activity of chitin [51]. The mutants D200A and D202A of Bc ChiA1, together with D170N of Th Chit42 proved to be active for chitinbiose oxazoline polymerization, and also for coupling reaction between Gal (β1→4) chitinbiose oxazoline and chitinpentaose at neutral pH. These mutants have retained the ability to catalyze transglycosylation reaction on natural COS. Such mutants could be considered as chitin transglycosylases.

Chitin synthase, a processive inverting enzyme of glycosyltransferase family 2, transfers GlcNAc from UDP-GlcNAc to preexisting chitin chains in reactions that are typically stimulated by free GlcNAc. This enzyme can be applied for synthesis of chitin oligosaccharides. By using a recombinant *Saccharomyces cerevisiae* chitin synthase with UDP-GlcNAc, GlcNAc_2_ was obtained as a major reaction product [143]. Formation of both COS and insoluble chitin was stimulated by GlcNAc_2_ and by *N*-propanoyl-, *N*-butanoyl-, and *N*-glycolyl-glucosamine.

### 7.5. Direct Fermentation of Raw Materials such as Crab and Shrimp Shells

The direct fermentation of raw materials such as crab and shrimp shells presents another opportunity in the production of chitin and COS. Chitin oligosaccharides were directly obtained from 1.5% shrimp head powder after 2 days of fermentation with protease- and chitosanase-producing *Bacillus cereus*TKU022 [144]. (GlcNAc)_2_, (GlcNAc)_4_, (GlcNAc)_5_, and (GlcNAc)_6_ were all identified in the culture supernatant. Even though the concentration of the products is still very low (0.3–201.5 μg/mL), these findings are meaningful in terms of direct production from raw materials.

### 7.6. Reactor Systems

Finally, the reactor system used should be considered to be a critical factor for efficiency and high yield in bioproduction. There are three types of reactors normally used in the preparation of chitosan oligosaccharides, including batch reactors, column reactors and ultrafiltration reactors [145]. Batch reactors represent the simplest method used for the enzymatic production of COS. This method has a few drawbacks, including limited reuse of reacting enzyme and continuous production of COS, difficulty in controlling molecular weight of COS, low yield and high cost.

Continuous preparation of COS is possible by packing immobilized enzymes into a column reactor that the substrate passes through. Though this method has several advantages over batch reactors, the poorer affinity of immobilized enzymes to chitosan substrate than that of free enzyme limits the activity of enzyme and the usage of column reactors in the commercial preparation of COS.

The ultrafiltration membrane reactor has been applied for overcoming the problems of the reusability of enzymes in batch reactors and the poor affinity of the substrate toward immobilized enzymes in column reactor systems, but suffers from the problem of membrane fouling [25,69]. A dual ultrafiltration membrane reactor system composed of a column reactor packed with immobilized enzymes and an ultrafiltration membrane reactor, has been invented and applied in COS production [146]. Using this reactor, chitosanase from *Bacillus pumilus* BN-262 continuously produced chitosan oligomers of DP 3~6 from chitosan (89% DD), free from any fouling problem.

## 8. Conclusions

Bioproduction of COS with enzymes and microorganismshas been studied for decades. However, the yield of bioproduction is still lower and the cost is higher than traditional chemical methods. Crude rather than pure enzyme preparations of chitinase, chitosanase, lysozyme, cellulase, protease, lipase, and pepsin were preferred for this purpose for the practical production of the oligosaccharides. The transglycosylation activity of chitinolytic enzymes was successfully exploited for the synthesis of desired chitin oligomers and their derivatives. Chitin deacetylase is also applicable to the preparation of oligosaccharides.

The direct degradation and separation of chitin from crab and shrimp shells and microbial cell walls presents a significant challenge in bioproduction of COS. The direct production of oligosaccharides from chitin and crab shells was achieved by using a combination of mechanochemical grinding and enzymatic hydrolysis. In case of chitosan oligosaccharides, salt forms are inevitably formed from aquatic reaction mixtures, which limit the application. However, the mechanochemical grinding of the substrate followed by enzymatic hydrolysis can circumvent this.

Breaking down the crystal structure of chitin and crab shells is necessary to improve enzymatic degradation of the substrate. Solubilization is the first choice for destruction of chitin crystallinity. Once solubilized in organic or heterogeneous solvent systems, the substrate must receive hydrolytic enzymes with special properties. Thus, screening high-potential enzymes that function in extreme environments is an important factor for the optimization of bioproduction.

The direct fermentation of raw biomaterials like crab and shrimp shells presents another opportunity in the production of chitin and COS. Even though the efficiency of this method is still very low, it is significant in terms of the direct production of COS from the row materials.

To overcome these inefficiencies, the optimization of a reactor system appropriate to enzymatic bioproduction is called for. A dual ultrafiltration membrane reactor system has been described that is capable of overcoming the problems of enzyme reusability in batch reactors as well as the poor affinity of the substrate toward immobilized enzymes in standard column reactor systems.
